# Supplementary material for: Flexible foraging behaviour increases predator vulnerability to climate change
Source: Nat Clim Chang. 2024 Feb 27;14(4):387–92. doi: 10.1038/s41558-024-01946-y (PMC11006620; doi:10.1038/s41558-024-01946-y)
Supplement: Supplementary file 1 — All supplementary material. [file 41558_2024_1946_MOESM1_ESM.pdf]

nature climate change

Supplementary information https://doi.org/10.1038/s41558-024-01946-y

Flexible foraging behaviour increases predator vulnerability to climate change

In the format provided by the authors and unedited

Table of Contents

Supplementary information I: Relations between environmental, realised and preference distributions. 2

Supplementary information II: Response of the realised body mass distribution to predator body mass and environmental distributions 3

Supplementary information III: Comparisons of model performances and diagnostics 4

Supplementary information IV: Environmental characteristics 5

Supplementary information V: Effect of temperature on the extinctions of basal species 7

Supplementary information VI: Effect of nutrient availability and predators' functional responses type on predictions about species coexistence. 8

Supplementary information VII: Sampling output and fish environments associations 11

Supplementary information VIII: Effect of considering different detection probabilities for prey in stomachs 12

Supplementary information IX. Definition of default model parameters 16

Supplementary information I: Relations between environmental, realised and preference distributions.

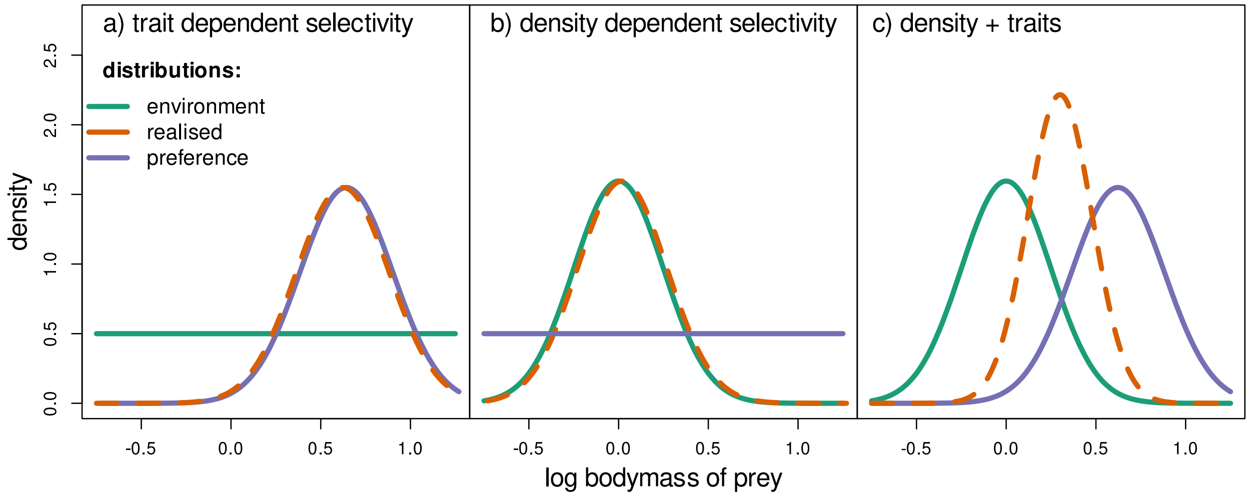

Figure SI 1: Illustration of the different fish prey body mass distributions. The environmental distribution (green) represents the distribution of prey body mass in the ecosystem, the realized distribution (dashed red) represents the body mass of the prey in a consumer stomach, and the preference distribution (blue) represents the selectivity of a consumer for a specific prey body mass. a) All of the log prey body masses are equally represented in the environment, so the distribution of prey body masses observed in a consumer's gut represents the body masses on which it actively foraged (its preference distribution) and predation is driven by trait selectivity only (hypothesis 1). b) The body mass distribution of the prey observed in the gut and in the environment are equivalent, so the prey consumed by the predator were entirely driven by encounter probabilities (i.e. a neutral process), implying no active selectivity over specific prey size classes (hypothesis 2). Panels a) and b) represent extreme scenarios while real-world data are more likely to be described by two different distributions, as in c) where the body mass distribution of prey observed in the stomach and in the environment differs, so that the consumer specifically forages on some prey body masses that are represented by the preference distribution. High values in the preference distribution represent body masses that are over-represented in fish stomachs compared to what is available in the environment.

Supplementary information II: Response of the realised body mass distribution to predator body mass and environmental distributions

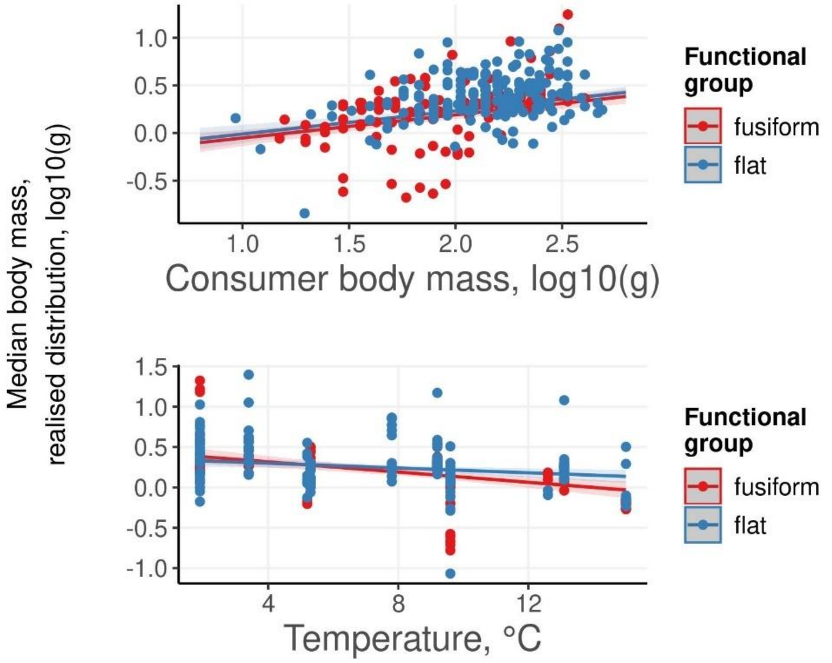

Figure SI 2: Response of the median body mass of the realised prey body mass distribution. Response to predator body mass (a), and temperature (b) for the two fish functional groups. Points represent non-transformed data across all productivity levels and lines present model predictions. Regression lines represent model's predictions for the median body mass of the realised distribution when all other covariates are considered. The shaded areas show the 95% confidence interval on the predicted values.

Table SI 1: Model coefficients. The model predicts the response of the median body mass of the realised distribution to predator traits and environmental gradients. Rhat values were all lower than 1.002. Number of samples was 4000.

Response of the median prey body mass of the realised distribution to			
Predictors	***Estimates***	***CI (95%)***	***Effective samples***
Intercept	-0.95723	-2.2867 – 0.3846	1809
Predator body mass	0.241989	0.1441 – 0.3399	3550
Temperature	0.036672	-0.0932 – 0.1734	1759
Productivity	0.305422	-0.1463 – 0.7688	1763
shapefusiform	0.083483	-0.0592 – 0.2259	2521
temperature:shapefusiform	-0.01697	-0.0333 – -0.0003	2406
temperature: resource availability	-0.0192	-0.0672 – 0.0263	1755
Observations	290		
R^2 Bayes	0.287		

Supplementary information III: Comparisons of model performances and diagnostics

Table SI 3: comparisons of model performences. The first row corresponds to the complete model, including all pairwise interactions (pred.BM:(resource availability), pred.BM:temperature, temperature:(resource availability) and temperature:shapefusiform). The second row corresponds to the model without the interaction between temperature and fish functional group. The third row corresponds to the model without fish functional group as a covariate. Δ elpd stands for differences in expected log pointwise predictive density (representing the goodness of model predictions) and Δ se its standard deviation (representing the uncertainty in the observed differences)

	Δ elpd	Δ se
Complete model	0.0	0.0
-(functional group) x temperature	-4.9	3.8
-functional group	-6.1	4.9

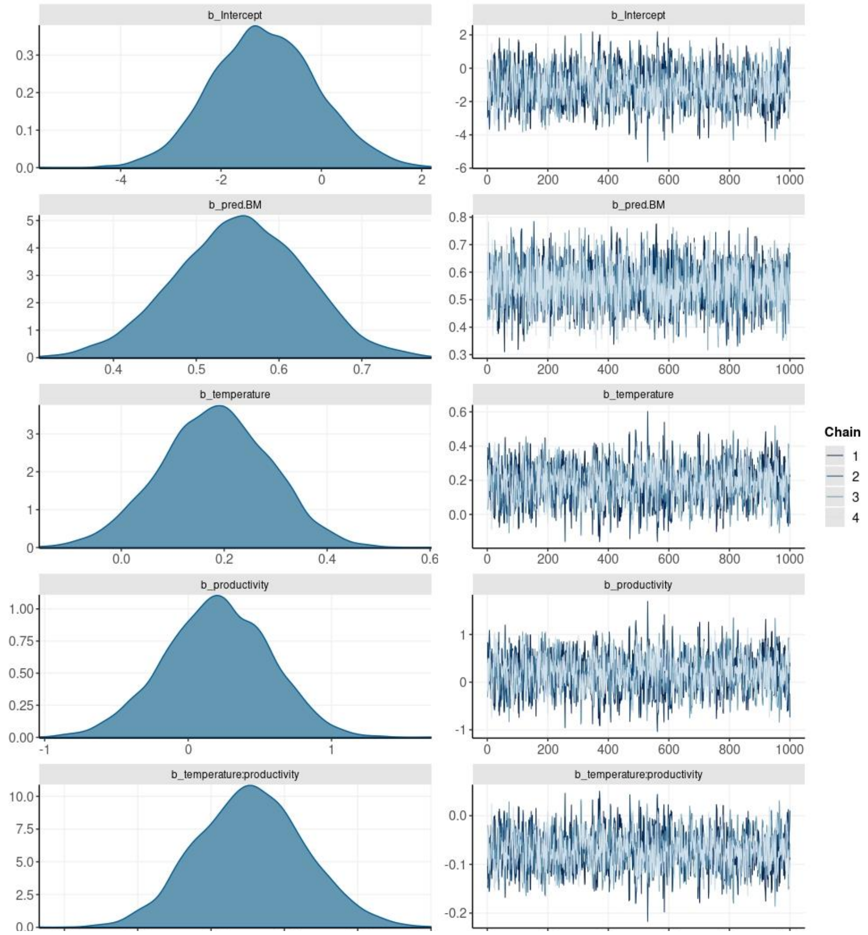

Figure SI 3: posterior distributions and chain mixing of the model without functional groups as a covariate

Supplementary information IV: Environmental characteristics

Overall, the different environments considered were characterised by two contrasted levels of productivity, leading to a bimodal distribution.

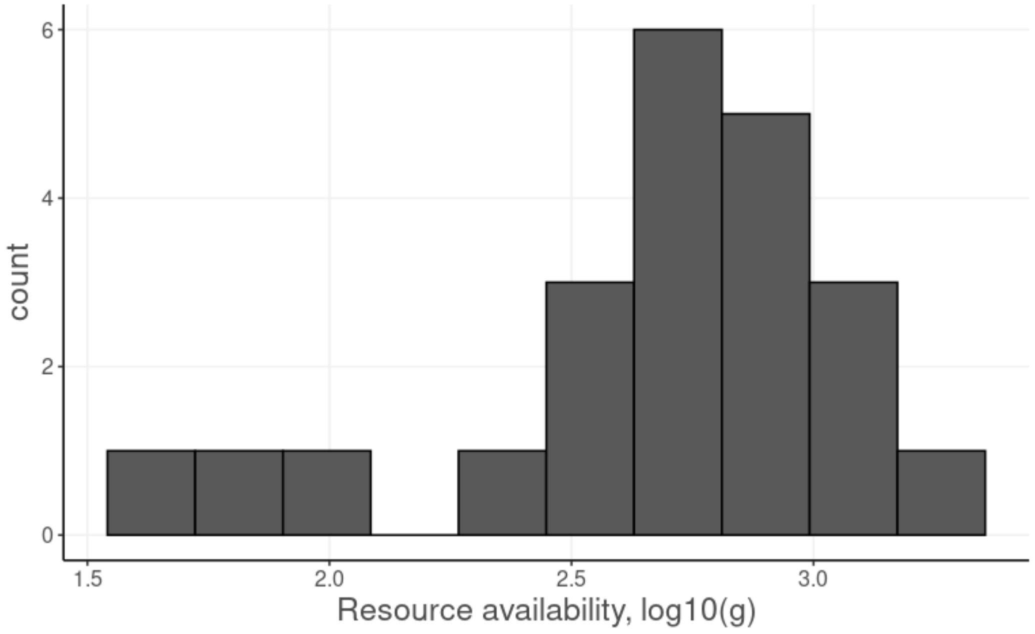

Figure SI 4: distribution of the resource availability for the different environments. Values are log10 transformed from grams (n = 22)

Associated to these differences, we observed that the body mass distribution of the prey species (median and standard deviation) was responding differently to temperature depending on productivity values (Figure SI II):

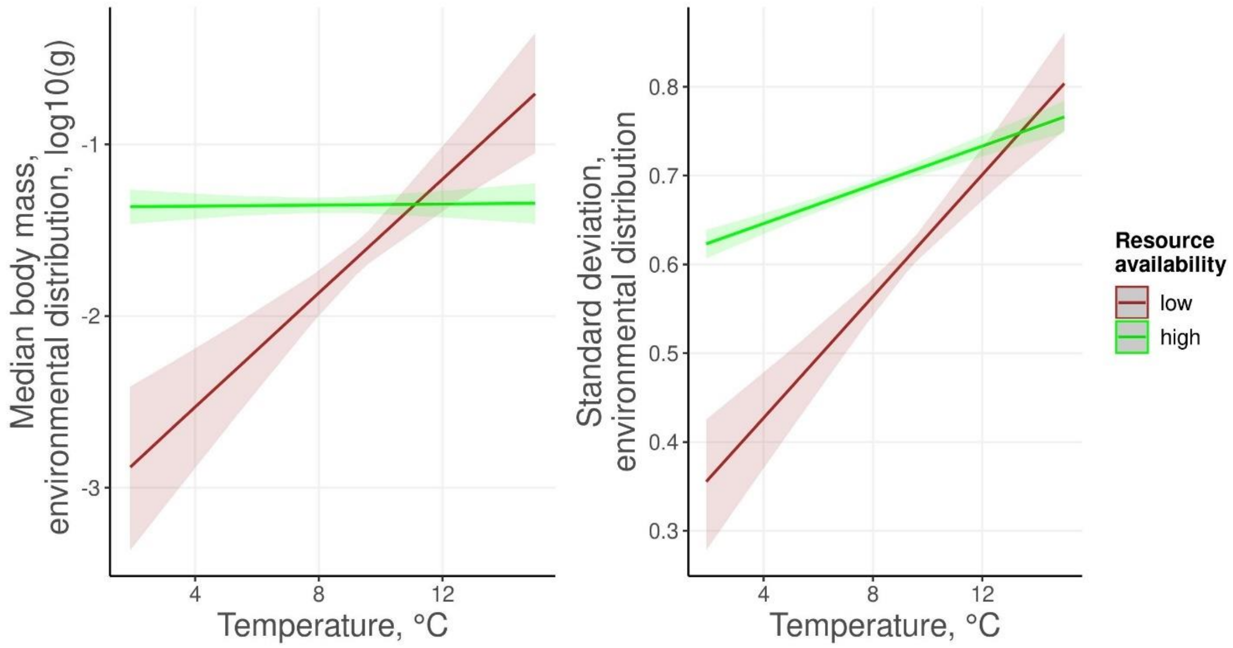

Figure SI 5: response of the body mass structure of the resource species (environmental distributions) to temperature and resource availability. Lines represents the model predictions and shaded areas their 95% confidence intervals

Table SI 4: model estimate for the prediction of median and standard deviation of the environment distributions. All Rhat values were lower than 1.006 indicating good chain mixing.

	**Median of BM**		**Standard deviation of BM**	
Predictors	*Estimates*	*CI (95%)*	*Estimates*	*CI (95%)*
Intercept	-7.54	-9.76 – -5.44	-0.45	-0.80 – -0.13
Temperature	0.56	0.35 – 0.78	0.09	0.06 – 0.13
Resource availability	2.29	1.55 – 3.08	0.39	0.28 – 0.51
Temperature: Resource availability	-0.21	-0.28 – -0.13	-0.03	-0.04 – -0.02
Observations	290		290	
R^2 Bayes	0.212		0.311	

Supplementary information V: Effect of temperature on the extinctions of basal species

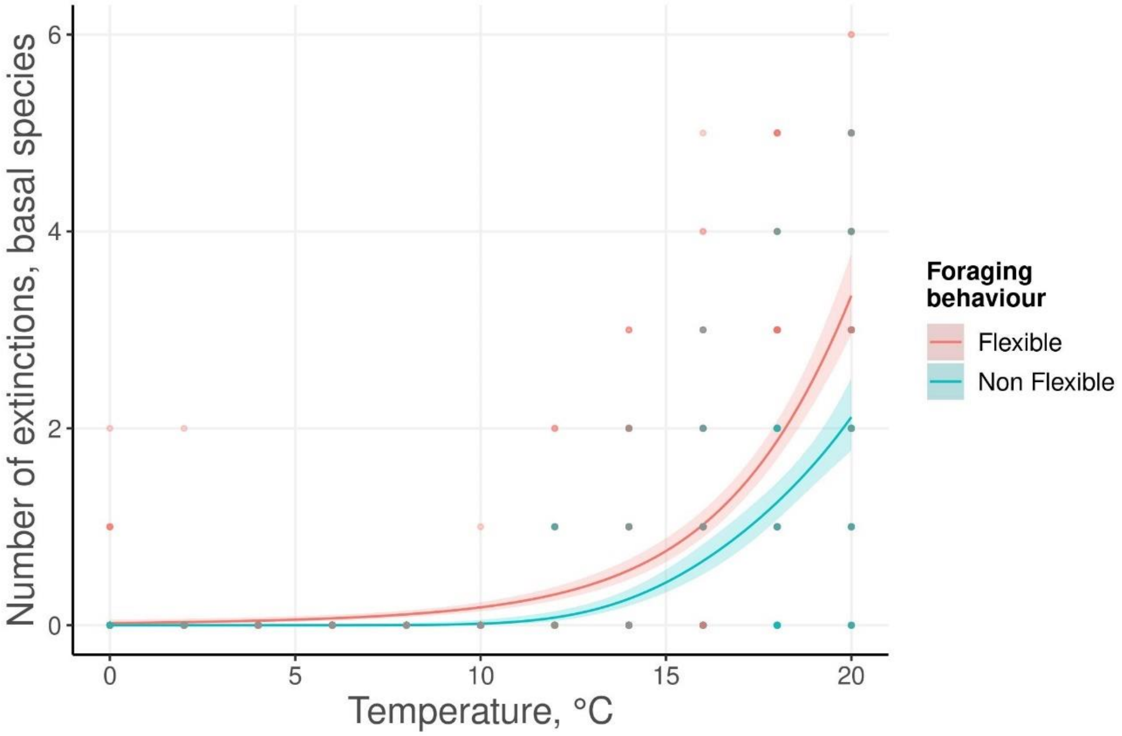

Figure SI 6: Number of basal species extinctions predicted by the model at different temperatures (out of an initial richness of 20 basal species). Points represent the number of observed extinctions for each simulation. The blue line represents the model output with adaptation of species diets to local temperature and productivity conditions considered, whilst the red line shows extinctions without allowing for this adaptation. The shaded areas show the 95% confidence interval on the predicted values. Predictions were estimated using a GAM with a binomial link function.

Supplementary information VI: Effect of nutrient availability and predators' functional responses type on predictions about species coexistence.

As maximum nutrient availability (variable S_i) and shape of the functional response (q) are not empirically informed, we analysed how sensitive to these two parameters model's predictions are. We varied S_i from 5 to 40 and q from 1 to 1.5 (Fig. SI 7). We can here observe that few differences occur between our two models at very low productivity levels while more extinctions occur when simulating communities with models incorporating flexible foraging behaviour. This is consistent with our experimental results showing an effect of temperature on consumers' diet at higher productivity levels only. Consistently to what is expected (Binzer et al. 2012, Binzer et al. 2016), higher productivity values balance the effect of temperature increase when foraging behaviour is not considered. However, when we incorporated the foraging behaviour described in our experimental results, we can see that this compensatory effect disappears. At higher productivity values we can see a more complex response from model incorporating foraging behaviour. This increase in number of extinctions observed when temperatures becomes very low could correspond to unstable dynamics explained by a relatively high inflow of energy to species from the higher trophic levels while their metabolic rates remains low, consistently with Gauzens et al. 2020 The type of the functional response used resulted in more variations on the number of extinctions observed, with differences in extinctions between the two different models increasing with q (Fig. SI. 8).

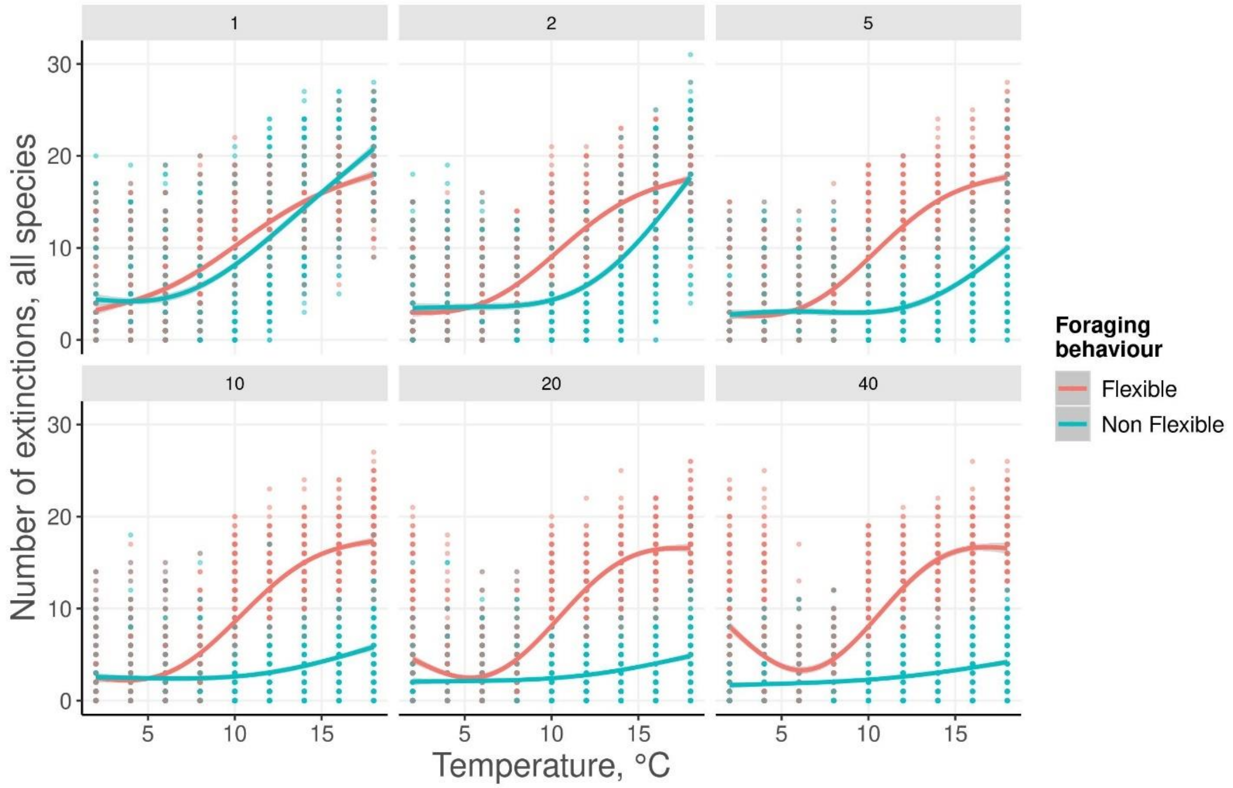

Figure SI 7: Effect of different levels of nutrient availability (variable S, values on top of the panels) on the number of extinctions predicted by the model. Simulations where ran with all hill exponent (q) values. Points represent the number of observed extinctions for each simulation. The blue line represents the model output with adaptation of species diets to local temperature and productivity conditions considered, whilst the red line shows extinctions without allowing for this adaptation. The shaded areas show the 95% confidence interval on the predicted values. Predictions were estimated using a GAM with a binomial link function.

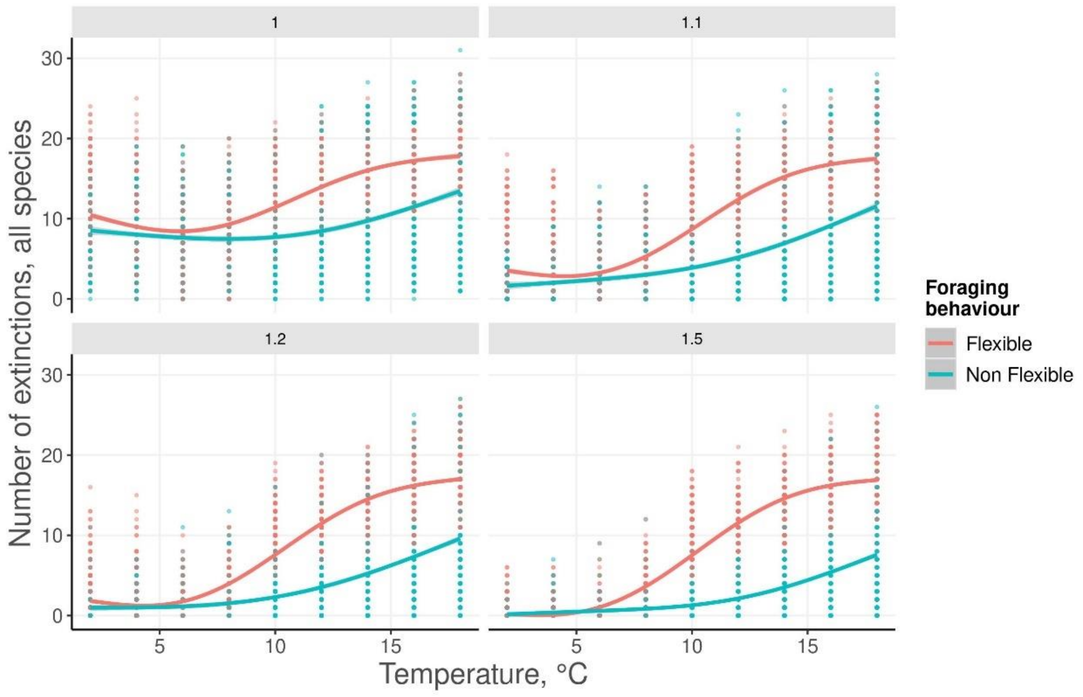

Figure SI 8: effect of the choice of functional response type (parameter q, values on top of the panels) on the number of extinctions predicted by the model. Simulations where ran for all level of maximum nutrient concentration (S). Points represent the number of observed extinctions for each simulation. The blue line represents the model output with adaptation of species diets to local temperature and productivity conditions considered, whilst the red line shows extinctions without allowing for this adaptation. The shaded areas show the 95% confidence interval on the predicted values. Predictions were estimated using a GAM with a binomial link function.

References:

Binzer Amrei, Guill Christian, Brose Ulrich and Rall Björn C. (2012), *The dynamics of food chains under climate change and nutrient enrichment*. Phil. Trans. R. Soc. B3672935–2944

Binzer, A., Guill, C., Rall, B.C. and Brose, U. (2016), *Interactive effects of warming, eutrophication and size structure: impacts on biodiversity and food-web structure*. Glob Change Biol, 22: 220-227

Gauzens, B., Rall, B.C., Mendonça, V. et al. (2020), *Biodiversity of intertidal food webs in response to warming across latitudes*. Nat. Clim. Chang. 10, 264–269

Supplementary information VII: Sampling output and fish environments associations

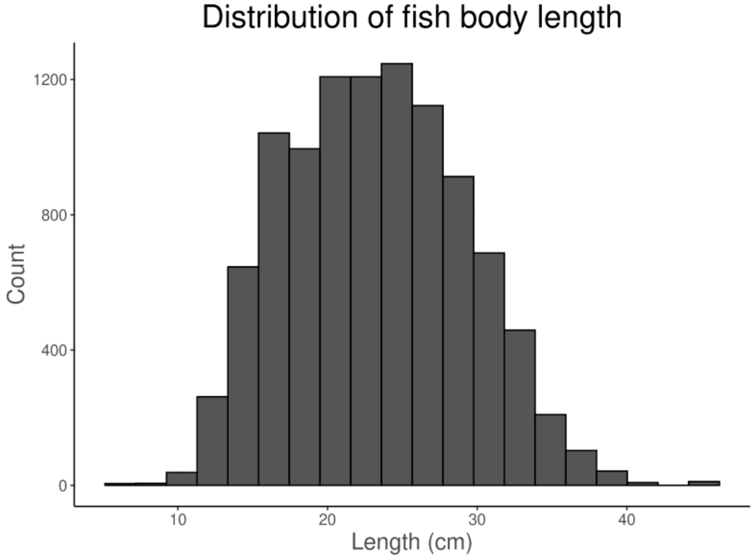

Figure SI VII.1: Distribution of fish body lengths (cm) in our database.

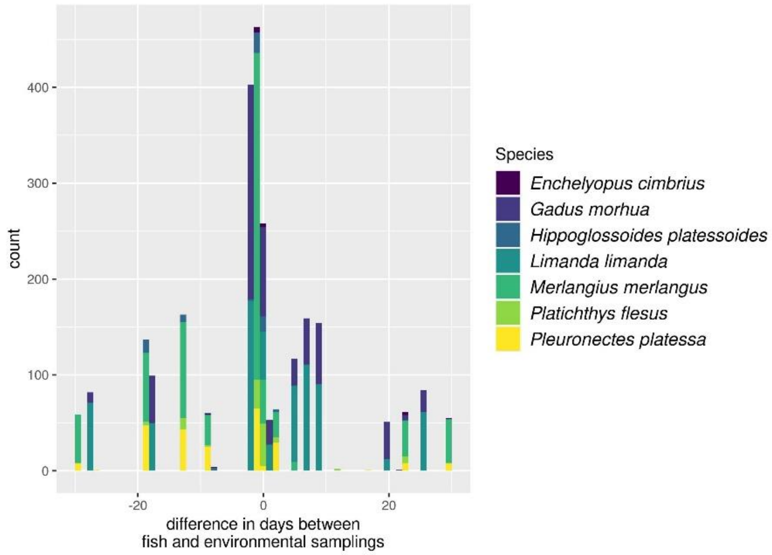

Figure SI VII.2: Distribution of temporal distance (days) between fish sampling and associated benthic samplings

Supplementary information VIII: Effect of considering different detection probabilities for prey in stomachs

As prey composed of soft tissues only are supposed to be less likely to be detected because of a faster digestion time, we corrected our observation by multiplying the abundance of species with hard body parts by 0.8. This was done to mirror the importance of these species that should persist longer in stomachs. As we are missing a general framework to properly describe how digestion time changes for the different species we used a unique correction factor that is a free parameter in our model (prey are either easy or difficult to digest, Table SI XI.2). We here present the results without this correction factor. Overall, these results remain qualitatively similar to the ones presented in the main text.

Figure SI VIII.1: Response of the median prey body mass of the preference distribution. Effect of (a), temperature (b) at different productivity levels. Preference values are based on data not corrected by prey digestibility. Points represent non-transformed data and lines present model predictions. The shaded areas show the 95% confidence interval on the predicted values.

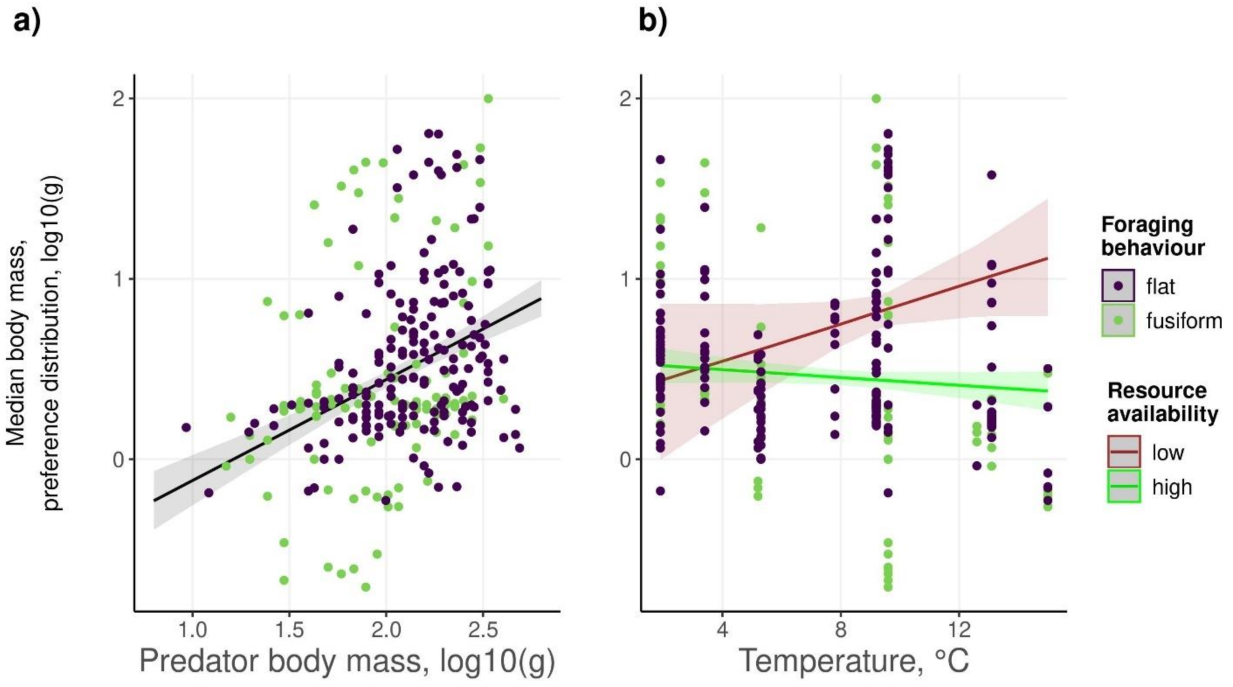

Table SI VIII.1: response of the realised distribution to predator body mass and environmental gradients when preferences are estimated with realised distributions non-corrected by prey digestibility. Rhat values were all lower than 1.005. Number of samples was 4000.

	**Response of the median prey body mass of the preference distribution to**		
Predictors	*Estimates*	*CI (95%)*	*Effective Samples*
Intercept	-1.32	-3.35 – 0.63	2071
Predator body mass	0.56	0.41 – 0.72	2252
Temperature	0.2	-0.0 – 0.41	1685
Resource availability	0.26	-0.43 – 0.97	1805
Temperature: Resource availability	-0.08	-0.15 – -0.01	1754
Observations	290		
R^2 Bayes	0.279		

We can observe that the absence of correction factor does not qualitatively change the trends observed for the preference distributions. We can only detect slight changes in the model estimates.

Table SI VIII.2: Classification of species' digestibility Classification of species' digestibility

Prey species	Class	Digestibility
Abra alba	Bivalvia	Hard
Aloidis gibba	Bivalvia	Hard
Amphicteis gunneri	Polychaeta	Easy
Amphipoda spp.	Malacostraca	Easy
Anaitides spp.	Polychaeta	Easy
Anthozoa spp.	Anthozoa	Easy
Aphia minuta	Actinopterygii	Hard
Aphroditidae spp.	Polychaeta	Easy
Arenicola marina	Polychaeta	Easy
Ascidiacea spp.	Ascidiacea	Easy
Astarte spp.	Bivalvia	Hard
Balanus spp.	Hexanauplia	Hard
Brada villosa	Polychaeta	Easy
Capitella capitata	Polychaeta	Easy
Carcinus maenas	Malacostraca	Hard
Cardium fasciatum	Bivalvia	Hard
Castalia punctata	Polychaeta	Easy
Clupea harengus	Actinopterygii	Hard
Corophium spp.	Malacostraca	Easy
Crangon crangon	Malacostraca	Hard
Cumacea spp.	Malacostraca	Easy
Mysidacea spp.	Malacostraca	Hard
Cyprina islandica	Bivalvia	Hard
Diastylis rathkei	Malacostraca	Easy
Disoma multisectosum	Polychaeta	Easy
Euchone papillosa	Polychaeta	Easy
Gastrosaccus spinifer	Malacostraca	Hard
Gobiidae spp.	Actinopterygii	Hard
Halicryptus spinolosus	Halicryptomorpha	Hard
Harmothoe imbricata	Polychaeta	Easy
Harmothoe spp.	Polychaeta	Easy
Hyperia galba	Malacostraca	Easy
Idothea spp.	Malacostraca	Hard
Isopoda spp.	Malacostraca	Hard
Limanda limanda	Actinopterygii	Hard

Prey species	**Class**	**Digestibility**
Macoma spp.	Bivalvia	Hard
Metridium senile	Anthozoa	Hard
Microdeutopus sp.	Malacostraca	Easy
Musculus spp.	Bivalvia	Hard
Mya truncata, Mya arenaria	Bivalvia	Hard
Mysis mixta	Malacostraca	Hard
Mytilus edulis	Bivalvia	Hard
Nemertea spp.	Nemertea	Easy
Nephthys spp.	Polychaeta	Easy
Nucula nitida	Bivalvia	Hard
Ophiura albida	Ophiuroidea	Hard
Other Decapoda	Decapoda	Hard
Other Gastropoda	Gastropoda	Hard
Other Polychaeta	Polychaeta	Easy
Pectinaria koreni	Polychaeta	Easy
Phaxas pellucidus	Bivalvia	Hard
Pherusa plumosa	Polychaeta	Easy
Phtisica marina, Caprella	Malacostraca	Easy
Pisces spp.	Actinopterygii	Hard
Pleuronectiformes spp.	Actinopterygii	Hard
Polydora sp.	Polychaeta	Easy
Pomatoschistus minutus	Actinopterygii	Hard
Priapulus caudatus	Priapulida	Easy
Saxicava arctica	Bivalvia	Hard
Scoloplos armiger	Polychaeta	Easy
Spionidae spp.	Polychaeta	Easy
Terebellides stroemi	Polychaeta	Easy
Thyonidium pellucidum	Holothuroidea	Hard

Supplementary information IX. Definition of default model parameters

Table SI IX.1: values and units of variables as set by default with the ATNr the package for the unscaled with nutrient version

Variable (units)	parameter used	values
b_{ij} $(m^2.s^{-1})$	b_0	50
	b_1	$\mathcal{N}(0.15,0.03)$
	b_2	$\mathcal{N}(0.47,0.04)$
	E_a	-0.38
h_{ij} (s)	h_0	0.4
	h_1	$\mathcal{N}(-0.66,0.02)$
	h_2	$\mathcal{N}(-0.48,0.03)$
	E_h	0.26
c_i (s)	c	$\mathcal{N}(0.8,0.2)$
K_{np} $(g.m^{-2})$	k	$\mathcal{U}(0.1,0.2)$
v_{np} $(unitless)$	v	$\mathcal{U}(1,2), \mid \sum_n (v_{np} = 1)$
S_n $(g.m^{-2})$	s	$\mathcal{N}(10,2)$
r_p (s^{-1})	r_1	-0.25
	E_r	-0.84
X_i $(J.s^{-1})$	x_0	0.138 (i is a plant)
	x_0	0.314 (i is an animal)
	x_1	-0.25
	E_x	-0.69
q_i	-	0.2
D	-	0.25
